# From Molecules to Mind: The Critical Role of Chitosan, Collagen, Alginate, and Other Biopolymers in Neuroprotection and Neurodegeneration

**DOI:** 10.3390/molecules30051017

**Published:** 2025-02-22

**Authors:** Weronika Kruczkowska, Julia Gałęziewska, Katarzyna Helena Grabowska, Piotr Gromek, Karolina Czajkowska, Maciej Rybicki, Mateusz Kciuk, Karol Kamil Kłosiński

**Affiliations:** 1 Department of Functional Genomics, Faculty of Medicine, Medical University of Lodz, Zeligowskiego 7/9, 90-752 Lodz, Poland; 2 Department of Biomedicine and Experimental Surgery, Faculty of Medicine, Medical University of Lodz, Narutowicza 60, 90-136 Lodz, Poland; 3 Department of Molecular Biotechnology and Genetics, University of Lodz, Banacha 12/16, 90-237 Lodz, Poland; 4 Biomaterials Research Laboratory, Faculty of Medicine, Medical University of Lodz, Narutowicza 60, 90-136 Lodz, Poland

**Keywords:** alginate, biopolymers, chitosan, collagen, neurodegeneration, neuroprotection

## Abstract

Neurodegenerative disorders present significant therapeutic challenges, particularly due to the complex nature of drug delivery to the central nervous system. This review investigates the applications of various biopolymers in neuroprotection and their potential role in treating neurodegeneration. We present a critical analysis of natural and synthetic biopolymers, focusing primarily on chitosan, fish collagen/gelatin, and alginate as key therapeutic agents. The review examines the fundamental mechanisms of brain development and neurodegeneration, establishing a framework for understanding how these biopolymers interact with neural tissues. By analyzing recent experimental studies, we evaluate the effectiveness of different biopolymer-based delivery systems in crossing the blood–brain barrier and their subsequent neuroprotective effects. Additionally, promising materials, including lignin, poly lactic-co-glycolic acid, and glucose-modified bovine serum albumin/procyanidin complexes, are briefly explored to provide a comprehensive overview of current developments in the field. Our analysis reveals that biopolymer-based approaches offer unique advantages in both neuroprotection and drug delivery, potentially opening new avenues for treating neurodegenerative conditions. This review synthesizes current knowledge and identifies promising directions for future research in biopolymer-based therapeutic strategies.

## 1. Introduction

The progressive and gradual loss of neurons is recognized as a fundamental pathological mechanism underlying neurodegenerative disorders (NDs). These disorders are chronic, progressive conditions characterized by the selective and symmetrical degeneration of neurons within motor, sensory, or cognitive systems [1,2]. Prominent examples of NDs include Alzheimer’s disease (AD), Parkinson’s disease (PD), Huntington’s disease (HD), and amyotrophic lateral sclerosis (ALS) [3]. Collectively, NDs, particularly AD and PD, affect over 60 million individuals globally and rank as the seventh leading cause of mortality worldwide, posing significant therapeutic challenges for contemporary clinicians [4]. The incidence of NDs is notably escalating. For instance, between 1990 and 2016, the global prevalence of dementia more than doubled, reflecting a 117% increase in cases during this period [5].

NDs were first described in the early 19th century. James Parkinson provided one of the earliest detailed accounts of an ND in his seminal work, An Essay on the Shaking Palsy, published in 1817. This condition was subsequently named PD in his honor [6]. In contrast, AD was first described in 1906 by Alois Alzheimer, who presented the case of Auguste Deter, a patient exhibiting progressive memory impairment and other neurological symptoms [7]. AD accounts for approximately 80% of dementia cases, which translates to nearly 24 million individuals affected globally. It is noteworthy that the term “neurodegenerative diseases” became widely adopted only in later years, coinciding with advancements in neurology and neuroscience [8,9].

NDs are a highly heterogeneous group of conditions in terms of their pathophysiology. The precise mechanisms underlying their development remain incompletely understood. One of the primary, though not exclusive, contributors to NDs is the aggregation and accumulation of misfolded proteins within or around neural cells, which is a phenomenon referred to as the “conformational disease” concept [10]. Neurodegeneration can also occur as a consequence of axonal transport defects, which are present in the majority of the previously mentioned disease entities [3]. The role of inflammation and the resulting production of reactive oxygen species (ROS) in neuronal damage should not be underestimated. Microglia and resident macrophages play a pivotal role in these processes, contributing to the neuroinflammatory response and oxidative stress that exacerbate neuronal injury [11,12].

Although each ND exhibits a distinct clinical course and specific manifestations, a common set of symptoms can be identified across many of these conditions. NDs are conventionally categorized into two main groups: those with a predominantly motor component, such as PD and HD, and those characterized primarily by cognitive decline, including AD, Lewy body dementia (LBD), and Frontotemporal Dementia (FTD) [13,14]. It is important to note that no ND is entirely devoid of either motor or cognitive impairments. The most common motor-related symptoms include bradykinesia, rigidity, and resting tremors [15]. In contrast, there is a loss of cognitive function and memory impairment, which is almost always followed by apathy and anxiety [16,17]. It should also not be ignored that all NDs, regardless of their classification, can induce mood disorders, even leading to depression. They can cause the appearance of negative symptoms such as delusions and hallucinations. In some cases, insomnia, olfactory, and/or appetite disturbances are observed [15,18,19].

NDs pose a significant challenge to healthcare systems. Currently, these conditions are incurable and chronic, and lead to irreversible, progressive deterioration in patients’ quality of life. They predominantly affect the elderly, who often present with multiple comorbidities. As life expectancy continues to increase, the prevalence of NDs is expected to rise, impacting an even greater number of individuals in the future [20]. Additionally, the rising prevalence of NDs will further strain the financial resources allocated for the treatment of an already challenging aging population. In the United States alone, the annual cost of treating PD exceeds USD 52 billion [21]. The substantial cost of therapy, the high prevalence, and the complexity of managing NDs have driven research efforts toward the development of alternative therapeutic approaches, like the utilization of biopolymers. Biopolymer-based delivery systems offer a potentially cost-effective alternative to traditional therapeutics. Derived from abundant natural sources, these systems can also reduce drug administration frequency through controlled release mechanisms. This combination of readily available materials and less frequent dosing contributes to lower overall treatment costs [22,23,24,25].

The distinct benefits of biopolymers have made them particularly promising options. Their natural origin or biomimetic design, which lowers inflammation and immunological reactions in neural tissues, accounts for their exceptional biocompatibility [26]. Direct access to impacted brain regions is made possible by the intentional engineering of numerous biopolymers to penetrate the blood–brain barrier by altering their surfaces or making use of natural transport processes [27,28]. Furthermore, these materials provide versatile drug delivery capabilities by enabling the exact temporal and spatial release of therapeutic drugs through regulated degradation rates and stimuli-responsive behavior. These synthetic or natural materials are particularly well suited for treating the intricate pathology of NDs because they can be designed to minimize systemic exposure and related adverse effects while maintaining therapeutic medication concentrations for prolonged periods of time [29,30,31].

Current therapeutic approaches for neurodegenerative disorders (NDs) remain insufficient, with limited efficacy and significant side effects, creating an urgent need for innovative treatment strategies. The development of biopolymer-based drug delivery systems for neuroprotection has made significant advances, as traditional non-biopolymer delivery systems often face limitations in achieving targeted and sustained drug release within the brain, with the blood–brain barrier (BBB) continuing to pose a significant obstacle [32,33]. Natural biopolymers such as chitosan, collagen, and alginate have emerged as promising candidates for neurodegenerative disorder treatment due to their inherent biocompatibility, biodegradability, and ability to be modified for enhanced BBB penetration, offering the potential for the sustained and controlled release of therapeutic agents while reducing systemic exposure and minimizing side effects [30,34]. A critical limitation is the lack of comprehensive comparative studies evaluating the efficacy and safety profiles of different biopolymers across various neurodegenerative contexts. Understanding the specific advantages and disadvantages of these biopolymers, along with the standardization of preparation methods and characterization techniques, is crucial for tailoring therapeutic strategies to individual disease profiles and ensuring reproducible clinical outcomes [34,35]. The complexity of biopolymer behavior in physiological environments, coupled with the unique challenges presented by the CNS, necessitates a deeper understanding of their interaction with neural tissues and their long-term effects on cellular functions [36,37]. Understanding these specific advantages and disadvantages is crucial for tailoring therapeutic strategies to individual disease profiles [38], and a deeper understanding of these limitations is essential to improve the design and application of biopolymer-based therapies [39]. Given these challenges in current ND treatments and the promising properties of biopolymers, our research focuses on investigating the mechanisms of action of drug delivery systems utilizing chitosan, collagen, and alginate for the neuroprotection and the treatment of NDs, aiming to address these fundamental limitations in the field [40,41].

## 2. Brain Development, Neurodegeneration, and Current Treatment

Understanding the complex relationship between brain development and neurodegeneration is crucial for developing effective treatments for NDs, such as AD and PD. Recent research suggests that the origins of these disorders may lie in early neural development, indicating that factors influencing brain formation could predispose individuals to NDs later in life [42].

Brain development is a complex and dynamic process that occurs both during prenatal and postnatal stages. It is characterized by distinct mechanisms essential for the formation of the brain’s structure and function. Prenatal brain development begins as early as the third week of gestation, with the formation of the neural plate, which subsequently folds to form the neural tube. By the eighth week, this structure has differentiated into three primary brain vesicles: the forebrain, midbrain, and hindbrain [43]. Neurogenesis typically occurs between weeks 4 and 20 of gestation. During this period, the majority of neurons are generated and migrate to their designated locations within the brain. By approximately 29 weeks of gestation, most neuronal migration is complete [43,44,45]. Following neuronal migration, synaptogenesis occurs as neurons begin to form synaptic connections. This process is characterized by a substantial increase in synapse density. The fetal brain undergoes significant growth, with an average increase in brain volume of approximately 2.3 mL per day during the third trimester (weeks 20–40). Additionally, at around week 25, myelination begins, involving the production of myelin sheaths around axons, which enhances the efficiency of signal transmission [44,46,47].

Postnatal brain development continues extensively after birth, with different regions maturing at varying rates. Synapse formation and subsequent pruning, which are processes that enhance neural connections, occur rapidly during early life, peaking around the age of five. Pruning refers to the elimination of excess neurons and synapses, facilitating more efficient neural networks. Sensory and motor regions develop, along with early language acquisition, followed by the emergence of fundamental emotional control circuits, which lay the foundation for social interactions. Myelination continues throughout development and adolescence, improving brain efficiency, particularly in white matter pathways. Experience also shapes brain connectivity, refining neural circuits. Executive functions, including decision-making and impulse control, emerge as the prefrontal cortex matures. During this time, the brain undergoes significant changes in both size and connectivity, reaching approximately 90% of its adult volume by age six. These developmental changes are influenced by both genetic and environmental factors [48,49].

NDs are characterized by the progressive loss of the neuronal structure and function. Clinically, these conditions often manifest decades after the onset of initial pathological changes. For instance, in AD, the deposition of beta-amyloid plaques can begin up to 20 years before the onset of clinical symptoms, suggesting that the origins of these diseases may lie in early developmental processes. Some studies propose that genetic alterations associated with NDs could lead to an “at-risk normal brain” during development. While such brains may function normally in early life, they become increasingly susceptible to various stressors with age. This perspective frames NDs as late-onset neurodevelopmental diseases rather than purely degenerative conditions [42,50,51].

Traditionally, the therapeutic approach for NDs has focused on symptom management rather than halting disease progression. Current treatments for AD primarily include cholinesterase inhibitors, such as donepezil, rivastigmine, and galantamine. These drugs temporarily improve cognitive function by increasing acetylcholine levels but do not modify the underlying course of the disease [52]. There are also monoclonal antibodies that target beta-amyloid plaques to lessen their load in the brain [53]. In PD, levodopa, a precursor to dopamine, is initially beneficial but loses effectiveness with time [54]. Incorporating biopolymers into current therapeutic approaches offers promise for improving drug delivery and efficacy [55]. Alginate-based hydrogels, for instance, can facilitate the sustained release of conventional drugs like levodopa, potentially widening its therapeutic window and mitigating motor complications [56,57]. Likewise, chitosan-based nanocarriers are explored for their potential to deliver drugs across the blood–brain barrier for treating Alzheimer’s disease and can be used to create scaffolds to support cell growth and tissue formation. Furthermore, research is now exploring disease-modifying therapies using biopolymers, aiming to achieve long-term therapeutic benefits [58]. Disease-modifying treatments that may give long-term benefits are now being researched.

Other emerging therapeutic strategies under investigation include gene therapy, which aims to correct or compensate for genetic defects associated with NDs, and stem cell therapy, which focuses on repairing damaged tissues and modulating immune responses. This approach often involves the transplantation of mesenchymal stem cells or their extracellular vesicles [59], and immunotherapy, which targets specific proteins involved in neurodegeneration to improve neuronal survival and function [60].

The complex connection between brain development and neurodegeneration provides opportunities for biopolymer-based therapeutics. The structural resemblance of certain biopolymers, especially collagen, to components of the neural extracellular matrix offers significant potential for neural regeneration and protection [26]. Collagen scaffolds, for example, can effectively mimic the natural neural microenvironment, providing structural support and promoting cell growth [61]. Furthermore, the positive charge of chitosan enhances cellular adhesion and proliferation, further contributing to its suitability for neural tissue engineering and repair [62].

## 3. Drug Delivery to the Brain

One of the main challenges for pharmacological treatments involves delivering medications to the components of the brain and its surrounding environment. Advances in nanotechnology and a greater understanding of the BBB mechanics—the primary obstacle to drug transport efficiency—have accelerated the development of drug delivery to the brain [32]. The blood–brain barrier is a highly selective, semipermeable barrier crucial for maintaining central nervous system (CNS) homeostasis. It tightly regulates the exchange of molecules between the blood and brain tissue, protecting the CNS from harmful substances. Composed of endothelial cells interconnected by tight junctions, astrocyte end-feet, and pericytes, the BBB selectively permits the passage of small hydrophobic molecules, nutrients, ions, and some organic anions. Tight junctions are the primary functional elements, establishing the barrier’s selective permeability and restricting paracellular diffusion. Beyond chemical exchange, the BBB also protects the brain from peripheral immune events [63,64].

The BBB presents unique challenges due to the highly selective character of this structure. By creating a highly selective semipermeable barrier with microvascular endothelial cells (BMVECs), it isolates the central nervous system from the flowing blood [33]. Tight junctions are specialized structures that firmly hold cells together, preventing paracellular transport, which is the movement of materials between cells. These junctions are composed of transmembrane proteins such as occludins, claudins, and scaffolding proteins, which collectively form a seal that restricts the passage of ions, small molecules, and larger macromolecules. This selective permeability is crucial for maintaining homeostasis and safeguarding the central nervous system from potentially harmful substances, including toxins and pathogens [64,65]. However, drug delivery is severely compromised by this protective barrier, especially when it comes to therapeutic drugs meant to treat neurological conditions. Because of their size and polarity, almost all macromolecular medicines, such as gene therapies and monoclonal antibodies, and around 98% of small-molecule medications are unable to penetrate the BBB efficiently. A variety of transport systems, each with unique properties and roles, allow molecules to pass through the BBB. Very small and only lipophilic compounds (less than 400–500 Da) may usually passively diffuse across the membranes of endothelial cells. Drug delivery attempts are made more difficult by the BBB’s active efflux transporters, such as P-glycoprotein, which can pump some medications back into the bloodstream [64,66,67].

As mentioned, the BBB poses a significant obstacle to CNS drug delivery, limiting the passage of numerous therapeutic agents. Many potential CNS therapeutics exhibit physiochemical properties, such as large size or hydrophilicity, which prevent BBB permeation, while others are susceptible to gastrointestinal degradation upon oral administration. Biopolymers present a potential solution due to their biocompatibility, biodegradability, and structural versatility [68,69,70]. They can be engineered into nanoparticles (NPs) for drug encapsulation, thereby protecting the therapeutic content from degradation and facilitating BBB transport. A promising strategy for evading the BBB is intranasal delivery, which targets the neuro-olfactory epithelium. This non-invasive route enables direct drug delivery to the brain, minimizing systemic exposure and potentially accelerating therapeutic onset [70,71].

Carrier-mediated transport (CMT) is another crucial transport mechanism that facilitates the passage of specific endogenous molecules, such as glucose and amino acids, across the blood–brain barrier. This process relies on specialized transport proteins encoded by the solute carrier (SLC) gene family [72]. Approximately 300 transporter genes are involved in this process, encoding membrane-bound proteins that facilitate the movement of various substrates across biological membranes. SLC transporters mediate the movement of a variety of molecules, including amino acids, glucose, nucleotides, and sulfates [73,74,75].

Many chemicals can cross biological boundaries thanks to a complex cellular process known as receptor-mediated transcytosis (RMT) [76]. Transcytosis is a key mechanism for transporting molecules across the BBB, enabling the delivery of therapeutics to the central nervous system. It involves the movement of macromolecules from one side of a cell to another via vesicles [64,77]. Large molecules can pass through tissues and arrive at particular locations within the body through a process called transcytosis. Endocytosis is triggered by ligand binding to luminal receptors in brain endothelial cells during RMT. Before being exocytosed into the brain parenchyma via the abluminal membrane, the cargo passes through vesicles for intracellular trafficking and sorting [76,78]. Several macromolecules necessary for brain function, such as insulin, transferrin, insulin-like growth factors, low-density lipoproteins, and antibodies, are transported across the BBB by RMT [79].

**Figure 1:**
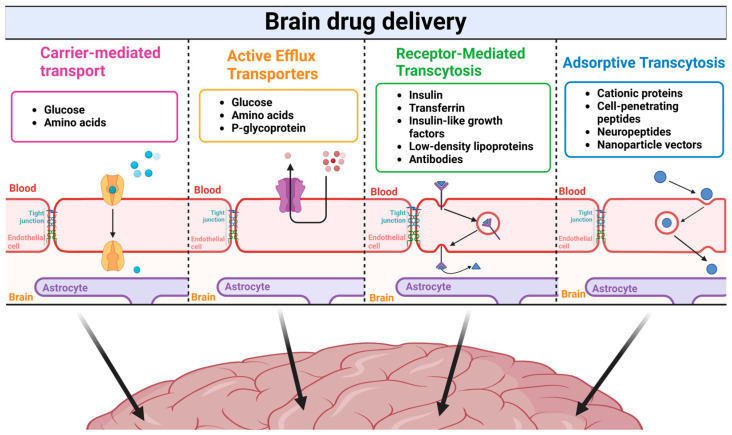
**Major transport mechanisms across the blood–brain barrier for drug delivery**. Carrier-mediated transport facilitates the movement of small molecules like glucose and amino acids through specific transporters (shown by light blue spheres). Active efflux transporters, including P-glycoprotein, regulate the transport of glucose and amino acids, often working against concentration gradients (represented by the purple spheres). Receptor-mediated transcytosis enables the transport of larger molecules such as insulin, transferrin, insulin-like growth factors, low-density lipoproteins, and antibodies through specific receptor recognition (depicted by triangles). Adsorptive transcytosis allows the passage of cationic proteins, cell-penetrating peptides, neuropeptides, and nanoparticle vectors through electrostatic interactions with the cell membrane (represented by dark blue spheres). Each of the transport type involves movement of different molecules, which are represented by the spheres or triangles and are present in the frames. Created in BioRender. Kciuk, M. (2025) https://BioRender.com/y65c011 (accessed on 23 January 2025).

Another type of transcytosis—adsorptive transcytosis (AMT)—occurs through electrostatic interactions between these molecules (such as cationic proteins, cell-penetrating peptides, neuropeptides, and nanoparticle vectors) and the negatively charged surface of brain capillary endothelial cells [64]. The process involves the binding of positively charged molecules to specific sites on the endothelial cell surface, followed by their uptake into the cell within small, membrane-bound compartments. These compartments then travel through the cell and release their contents on the opposite side, allowing the molecules to enter the brain tissue [80,81,82]. These mechanisms provide prospects for drug delivery techniques aimed at treating diseases of the central nervous system in addition to facilitating the entry of essential nutrients. Four principal mechanisms of drug transport across the blood–brain barrier are illustrated in Figure 1.

## 4. Types of Biopolymers and Their Neuroprotective Effect

**Figure 2:**
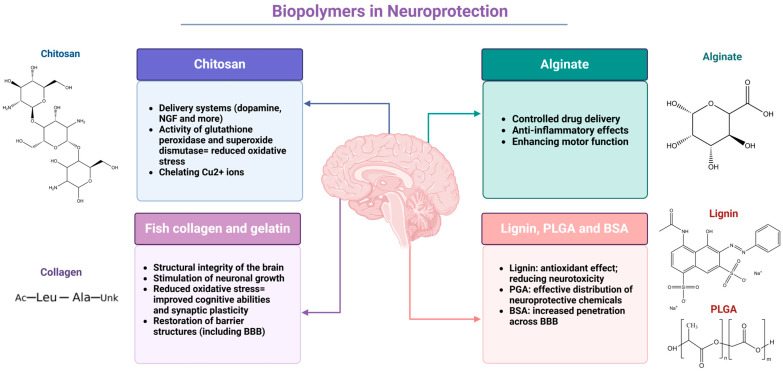
**Biopolymers and their neuroprotective mechanisms in brain health.** Chitosan demonstrates multiple neuroprotective functions, including neurotransmitter delivery (dopamine, nerve growth factor (NGF)), antioxidant effects through glutathione peroxidase and superoxide dismutase regulation, and Cu^2+^ ion chelation. In contrast, alginate contributes to controlled drug delivery and exhibits anti-inflammatory effects and motor function enhancement. Fish collagen and gelatin maintain brain structural integrity, stimulate neuronal growth, reduce oxidative stress for improved cognition, and restore barrier structures, including the blood–brain barrier (BBB). The lignin/poly(lactic-co-glycolic acid) (PLGA)/bovine serum albumin (BSA) combination provides complementary benefits: lignin acts as an antioxidant-reducing neurotoxicity, PLGA enables the efficient distribution of neuroprotective agents, and BSA enhances BBB penetration. Created in BioRender. Kciuk, M. (2025) https://BioRender.com/h27p913 (accessed on 16 February 2025).

The challenge of delivering therapeutic agents to the brain is primarily due to the presence of the BBB, which restricts the passage of most drugs. Recent studies have focused on utilizing biopolymers, such as chitosan and alginate, to enhance drug delivery systems aimed at treating NDs and promoting neuroprotection. This section reviews key experimental and clinical trial articles that highlight the effectiveness of these biopolymers in brain drug delivery and their potential applications in neuroprotection and neurodegeneration [27,83,84]. The summarized version of the neuroprotective effect of analyzed biopolymers is shown in Figure 2.

### 4.1. Chitosan

Chitosan, a biopolymer derived from chitin, offers promising health benefits. Its distinctive chemical structure, featuring amino and hydroxyl groups, enables it to interact with a variety of substances, including dietary fats. This interaction has the potential to reduce fat absorption in the gastrointestinal tract, thereby supporting weight management [85,86]. Furthermore, chitosan may also play a role in cholesterol regulation by interfering with the absorption of dietary cholesterol [87]. Its high charge density facilitates the interaction with blood components, stimulating platelet activation and fibrinogen adsorption, which are crucial steps in blood clotting [88]. These properties, coupled with its biocompatibility (the ability to interact with a living system without causing harmful effects) and biodegradability, make chitosan a promising candidate for use as a functional ingredient in dietary supplements or a great material for various medical applications [89,90].

Chitosan nanoparticles (CS NPs) have been widely studied as delivery vehicles for drugs like dopamine in treating neurodegenerative conditions like PD [91,92]. Aikaterini-Theodora et al. investigated chitosan-coated poly(lactic-co-glycolic acid) (PLGA) nanoparticles for the nasal delivery of ropinirole hydrochloride, demonstrating enhanced permeation through sheep nasal mucosa [93]. Additionally, studies have shown that modified CS NPs can facilitate gene delivery systems that provide neuroprotection in models of PD. For example, Yongyong et al. reported on CS NPs conjugated with nerve growth factor (NGF), which exhibited significant protective effects on dopaminergic neurons in vitro and in vivo [94]. CS NPs also provide a controlled and sustained release of dopamine while protecting it from premature oxidation. This was achieved through enhanced antioxidant enzyme activities, particularly glutathione peroxidase and superoxide dismutase, which help scavenge ROS [95]. The formulation and characterization of CS NPs loaded with the neuroprotective flavonoid derived from *Phyllanthus niruri* Linn demonstrated significant protective effects against oxidative stress in neuronal cells. This study emphasizes the sustained release profile of these nanoparticles, which results in reduced oxidative stress levels compared to the administration of the free drug [96]. Such formulations can be crucial in managing oxidative damage associated with NDs.

Additionally, chitosan itself has inherent neuroprotective properties, as it can chelate Cu^2+^ ions that would otherwise produce harmful hydroxyl radicals, causing lipid peroxidation. The biocompatibility, biodegradability, and mucoadhesive properties of chitosan also make it an excellent candidate for targeted drug delivery across the BBB [91]. This biopolymer shows promise as a neuroprotective agent and drug delivery vehicle for treating neurological disorders like AD. Its ability to cross the BBB makes it useful for delivering drugs and siRNA to the brain. CS NPs can be modified to target specific brain regions and improve drug absorption [97,98]. Studies have demonstrated chitosan’s potential to reduce amyloid plaque, deliver therapeutic agents, and protect neurons in AD models [99,100]. They have also been tested for the nasal delivery of different compounds (such as curcumin) with anti-depressant effects [101]. Wilson et al. explored CS NPs as a new delivery system for tacrine, an anti-AD drug. This study indicated that tacrine-loaded CS NPs improved drug bioavailability and therapeutic efficacy in a rat model, suggesting their potential to enhance pharmacokinetic properties in neurological treatments [102]. In another study, Gholizadeh et al. developed a smart thermosensitive chitosan hydrogel for the nasal delivery of ibuprofen, specifically targeting neurological disorders. Their formulation exhibited rapid gelation at physiological temperatures and significantly improved ibuprofen solubility, facilitating effective transport across human nasal epithelial cells [99]. This approach not only enhanced drug delivery but also provided a non-invasive method for treating conditions like migraines and other neurological ailments. The studies mentioned above are summarized in Table 1.

### 4.2. Fish Collagen and Gelatin

Collagen is a major structural protein. Collagen filaments provide structural support for all organs of the body, imparting strength, flexibility, and firmness. Biodegradability, absorbability, high biocompatibility, and porosity allow collagen to be a protein with multiple applications [103]. It is also worth pointing out that this biopolymer can be easily processed by both physical and chemical methods. Conversely, it should be mentioned that these processes negatively affect biocompatibility and may result in cytotoxicity [104]. Due to its versatility, collagen has been widely utilized across various industries, including food, cosmetics, pharmaceuticals, and biomedicine. However, ethical and religious concerns have led to a decreasing use of exogenous collagen, primarily derived from mammals. As an alternative, collagen sourced from fish has emerged as a promising substitute. Recent years have brought a significant increase in the consumption of fish products, resulting in a growing volume of byproducts that cannot be stored indefinitely. Consequently, this has led to escalating environmental pollution, particularly in marine ecosystems [103,105].

It is important to highlight that synthesis methods for fish collagen are simple, affordable, and highly efficient. The widespread production of this biopolymer would reduce the amount of accumulated fish waste, which would have a positive impact on the quality of the environment, especially the seas and oceans. In addition, it would be a promising substitute for mammalian-origin collagen use [103]. Recently, there has been a rising interest among researchers in the use of fish collagen in medical applications. The influence of collagen, particularly fish-derived collagen, in the treatment of angiogenic diseases is notable. However, its most promising application appears to be in the field of neurology [103,106].

The use of a collagen matrix in rats after head injuries has been shown to reduce the post-traumatic lesion volume and improve spatial memory. These findings highlight a clear neuroprotective effect, suggesting the potential for broad medical applications [107,108]. The positive influence of fish collagen on stem cells has been demonstrated, including the stimulation of neuronal growth [109]. For example, this biopolymer stimulated neural plate formation during embryonic development from pluripotent stem cells [110]. Moreover, it has been observed to accelerate and increase the efficiency of learning processes in aged mice. There is also a correlation between improved cognitive abilities and reduced oxidative stress damage to neural tissue using fish collagen in mice [109,111]. For instance, Smagin et al. investigated the aberrant expression of collagen gene family members in brain regions impacted by chronic agonistic interactions in male mice. Their results revealed that chronic social stress-induced significant changes in collagen gene expression, particularly in areas involved in mood regulation, such as the hypothalamus and hippocampus. The upregulation of these collagen genes may reflect alterations in the extracellular matrix (ECM), potentially contributing to behavioral psychopathologies akin to those observed in humans exposed to chronic stress [112]. Koizumi et al. conducted a pilot clinical study assessing the effects of collagen hydrolysates on the human brain structure and cognitive function. Their results suggested that dietary supplementation with collagen could have beneficial effects on cognitive health by potentially enhancing synaptic plasticity and neuronal health [113]. Collagen is essential for both the structural integrity and the functional aspects of brain health, according to these findings.

Gelatin is a protein that does not naturally occur in the environment. It is produced through the hydrolysis of native collagen and shares similar properties with gelatin derived from, for example, mammalian skin. However, gelatin derived from alternative sources exhibits weaker gelling properties and strength, as well as reduced stability compared to its mammalian counterpart [114,115,116]. The application of gelatin provides an optimal, amino acid-rich environment, which is essential for the restoration of barrier structures. It is also recognized for its use in the treatment of BBB leaks [117,118]. For instance, Kumosa et al. found that the gelatin coating of brain implants offers reduced inflammatory sequelae and long-term neuroprotective effects [117].

Additionally, gelatin has been shown to mitigate inflammation following central nervous system puncture wounds, suggesting its potential application in neurosurgery [117]. This is also evidenced by the fact that studies have reported strong neuroprotective effects of gelatin on SH-SY5Y neuroblastoma cells [118]. Similarly to mammalian-derived gelatin, fish gelatin also exhibits the ability to prevent ROS-induced cell death and subsequent functional impairment. This was demonstrated in a study using neuron-like PC12 cells exposed to hydrogen peroxide (H_2_O_2_)-induced oxidative stress [119]. Some positive protective effects against beta-amyloid-associated neurotoxicity (in AD) have been demonstrated, which provides a potential opportunity to use fish gelatin in the treatment of NDs. In addition, antimicrobial and antioxidant activities are also seen, which have been validated in vitro and in vivo [120]. The vast majority of studies on the neuroprotective properties of collagen, gelatin, and their components have been conducted on animal models. In contrast, human studies are limited, so further, more advanced clinical studies are needed to unambiguously confirm the beneficial properties of these substances in humans. The studies described above are summarized in Table 1.

### 4.3. Alginate

Alginates are natural polysaccharides derived predominantly from brown algae, with distinct structural and functional features that make them useful in a variety of applications, including food, medicines, and biomedicine. They are linear copolymers made up of two types of uronic acid monomers: D-mannuronate (M) and L-guluronate (G). The M/G ratio, which varies among alginate sources, significantly influences their properties. Alginates can form gels upon their interaction with divalent cations, with stronger gels produced when G blocks are present. They are soluble in water but can form hydrogels when exposed to calcium ions [121]. Alginates are biocompatible, suitable for biomedical applications, and have thickening properties in food products and industrial applications. Their properties can be influenced by source species, environmental conditions, and the M/G ratio. These characteristics can vary significantly based on these factors [122,123,124].

Recent studies have shown that alginate-based systems can offer neuroprotection through multiple mechanisms, including anti-inflammatory effects, controlled drug delivery, and structural support in various neurological conditions. In PD research, polymannuronic acid (PM) has shown particular promise by enhancing motor function, preventing dopaminergic neuronal loss, and modulating neurotransmitter levels. In contrast, polyguluronic acid (PG) exhibits distinct but complementary neuroprotective properties [125,126]. In spinal cord injury applications, alginate hydrogels serve as both scaffolds for tissue regeneration and delivery vehicles for therapeutic agents [126], with enhanced outcomes demonstrated when combined with human endometrial stem cells and curcumin-loaded PLGA nanoparticles [127]. The versatility of alginates is further exemplified by their ability to form specialized delivery systems, such as macrophage-derived nanovesicles containing sodium alginate and naloxone, for targeted delivery, which effectively reduces inflammation and neuronal apoptosis [128]. Studies have also demonstrated the efficacy of polyelectrolytically coated alginate systems for sustained drug delivery in retinal applications. Additionally, sodium alginate encapsulation has been shown to enhance the brain delivery of therapeutic compounds, such as probucol, resulting in reduced neuroinflammation and suppressed neurodegeneration [129,130]. Most recently, magnesium alginate formations have demonstrated unique properties for injectable applications, offering both neuroprotective effects through sustained magnesium ion release and enhanced oxidative stress protection in neural stem cells [131], suggesting broad potential for treating various neurological conditions. These diverse applications and positive outcomes across multiple neurological conditions highlight alginates’ versatility and effectiveness as a therapeutic platform in neurological medicine. Singh et al. synthesized and characterized alginate and sterculia gum-based hydrogels specifically for brain drug delivery applications. Their findings suggest that these hydrogels can effectively encapsulate therapeutic agents while providing a suitable environment for neuronal cells [132]. Furthermore, El Kheir et al. investigated the impact of simulated brain interstitial fluid flow on the release of chemokine CXCL12 from an alginate-based hydrogel using a novel 3D in vitro model. This study highlights how dynamic conditions can influence drug release profiles, which is essential for optimizing therapeutic interventions targeting neuroinflammation [133].

Alginates have emerged as versatile biomaterials in neurological medicine, offering unique advantages in drug delivery, tissue regeneration, and neuroprotection through their tunable properties and biocompatibility, although further research is needed to fully understand their long-term safety, optimize their properties for specific applications, and translate these promising preclinical findings into clinical trials. They have demonstrated effectiveness across various neurological conditions, combined with ongoing developments in specialized formulations, which positions them as promising therapeutic platforms for advancing neurological treatments. The studies mentioned above are summarized in Table 1.

### 4.4. Other Biopolymers

Lignin, found in the cell walls of plants, is a complex organic polymer with antioxidant characteristics. Recent investigations have shown that lignin–carbohydrate compounds have strong neuroprotective properties against oxidative stress. For example, one study found that lignin might reduce neurotoxicity caused by bisphenol A in zebrafish models through antioxidant properties and the modulation of apoptotic pathways by the regulation of proteins like B-cell lymphoma 2 (BCL-2) and Bcl-2-associated X protein (BAX) expression, thereby protecting neuronal integrity from excitotoxicity [134,135,136].

PLGA is a biodegradable polymer often utilized in medication delivery systems. Its use in neuroprotection has been investigated through the encapsulation of therapeutic drugs that pass the BBB. PLGA nanoparticles have exhibited the effective distribution of neuroprotective chemicals while preserving biocompatibility and stability, hence boosting their potential in treating neurodegenerative diseases [137]. Yusuf et al. targeted polysorbate-80-coated PLGA thymoquinone nanoparticles for the treatment of Alzheimer’s disease and demonstrated that PLGA nanoparticles, when properly coated with P-80, serve as an effective delivery system for obtaining therapeutic compounds across the blood–brain barrier for neuroprotection [138].

Recent studies have focused on glucose-modified bovine serum albumin (BSA) nanoparticles that contain procyanidin C-1, a natural polyphenolic compound. These nanoparticles were developed to increase medication penetration across the BBB by targeting glucose transporter 1 (GLUT1), which is responsible for glucose supply to the brain and other organs. This study found that nanoparticles not only crossed the BBB but also improved cognition in AD models by lowering amyloid-beta deposition and tau phosphorylation. They stimulated the phosphoinositide 3-kinase (PI3K)/protein kinase B (AKT) pathway and inhibited neuroinflammatory responses mediated via the NOD-like receptor family pyrin domain containing 3 (NLRP3)/caspase-1/interleukin-1 beta (IL-1β) pathway [139]. The studies described above are summarized in Table 1.

**Table 1:** **Experimental studies of biopolymers in neurological applications**. This table summarizes key experimental and clinical research studies investigating various biopolymers (chitosan, fish collagen, fish gelatin, alginate, lignin, poly(lactic-co-glycolic acid) (PLGA), and modified bovine serum albumin (BSA)) in neurological applications. Each entry details the specific study’s focus, main findings, and corresponding reference numbers, highlighting the diverse approaches and outcomes in neuroprotection, drug delivery, and treatment of neurological conditions.

Study Type	Biopolymer	Study Focus	Study Details	Key Findings	Authors
In vitro	Chitosan	Nasal delivery of ropinirole hydrochloride using chitosan-coated PLGA nanoparticles	Used ex vivo sheep nasal mucosa model	Enhanced permeation through sheep nasal mucosa	A.T. Chatzitaki et al. [93]
In vitro/In vivo	Chitosan	NGF-conjugated chitosan nanoparticles	Used ex vivo sheep nasal mucosa model	Protective effects on dopaminergic neurons in PD models	Y. Xue et al. [94]
In vitro	Chitosan	Neuroprotective flavonoid-loaded chitosan nanoparticles	Cell culture study using neuronal cell lines	Reduced oxidative stress in neuronal cells	G. Rajamanickam et al. [96]
In vivo	Chitosan	Tacrine delivery system	Rat model study with behavioral and biochemical analysis	Improved drug bioavailability in AD rat model	B. Wilson et al. [102]
In vitro	Chitosan	Thermosensitive hydrogel for ibuprofen delivery	Human nasal epithelial cell culture study	Enhanced solubility and transport across nasal epithelial cells	H. Gholizadeh et al. [99]
In vitro	Chitosan	Dopamine delivery system	Cell culture study evaluating antioxidant enzyme activities	Reduced oxidative stress through enhanced antioxidant enzyme activities; sustained release profile	A. Ragusa et al. [95]
In vivo	Chitosan	Luteolin-loaded chitosan nanoparticles	Animal model study of AD with behavioral assessment	Effective brain targeting in AD model; demonstrated antioxidant and anti-inflammatory effects	H. Abbas et al. [100]
In vitro	Chitosan	Curcumin-loaded guanidine–chitosan hydrogel	Animal study evaluating anti-depressant effects	Enhanced nasal delivery with anti-depressant effects; improved bioavailability	X.-J. Qi et al. [101]
In vivo	Fish collagen	Collagen matrix for head injuries	Rat model study with traumatic brain injury	Reduced post-traumatic lesion volume	S.S. Shin et al. [107]
In vivo	Fish collagen	Collagen matrix for head injuries	Mouse model study with Morris water maze testing	Improved spatial memory	J.-H. Chen et al. [108]
In vivo	Fish collagen	Effect on collagen gene expression	Mouse model study examining chronic social stress	Changes in mood regulation areas due to chronic stress	D.A. Smagin et al. [112]
Clinical	Fish collagen	Clinical study of collagen hydrolysates	Human trial with cognitive testing and brain imaging	Enhanced synaptic plasticity and cognitive function	S. Koizumi et al. [113]
In vivo	Gelatin	Impact of gelatin coating on brain implants and tissue response after acute brain injury compared to uncoated stainless-steel implants	Rat model study with histological analysis	Gelatin coating on brain implants significantly accelerated BBB healing after injury, modulated the inflammatory response and had minimal impact on neurons and astrocytes	L.S. Kumosa et al. [117]
In vitro	Fish gelatin	Effect on neuroblastoma cells	Cell culture study using neuroblastoma cell line	Neuroprotective effects on SH-SY5Y cells	S. Wang et al. [118]
In vitro	Fish gelatin	Protection against oxidative stress	Cell culture study using PC12 cells	Prevented ROS-induced cell death in PC12 cells	L. Xiao et al. [119]
In vitro	Fish collagen	Neural plate formation from stem cells	Stem cell culture study	Enhanced the formation of the neural plate during embryonic development from pluripotent stem cells	M. Iwashita et al. [110]
In vivo	Fish collagen	Learning processes in aged mice	Aged mouse model with cognitive testing	Improved learning efficiency and cognitive abilities in aged mice; reduced oxidative stress damage	X. Pei et al. [109]
In vivo	Alginate	Polymannuronic acid in PD	Mouse model study with behavioral testing	Enhanced motor function	Z.-R. Du et al. [125]
In vivo	Alginate	Polymannuronic acid in PD	Animal model of PD with histological analysis	Prevented dopaminergic neuronal loss	S. Grijalvo et al. [126]
In vivo	Alginate	Combined with stem cells and curcumin–PLGA	Rat model study with functional recovery assessment	Enhanced outcomes in spinal cord injury	A. Ai et al. [127]
In vitro	Alginate	Macrophage-derived nanovesicles	Cell culture study using primary neurons	Reduced inflammation and neuronal apoptosis	X. Liu et al. [128]
In vitro	Alginate	Magnesium alginate formulations	Neural stem cell culture study	Enhanced oxidative stress protection in neural stem cells	J.C. Mamo et al. [130]
In vitro	Alginate	CXCL12 release from hydrogels	Three-dimensional in vitro model with simulated brain conditions	Dynamic release profiles in brain interstitial fluid	El Kheir et al. [133]
In vivo	Alginate	Polyelectrolytically coated intraocular systems	Animal model of retinal degeneration	Enhanced photoreceptor survival in retinal degeneration; sustained drug delivery	F.S.Y. Wong et al. [129]
In vitro	Alginate	Alginate–sterculia gum hydrogels	Cell culture study with neuronal cells	Effective drug encapsulation and suitable environment for neuronal cells	B. Singh et al. [132]
In vivo	Lignin	Protective effects of lignin–carbohydrate complexes against bisphenol A-induced neurotoxicity in zebrafish	Zebrafish model study	Enhanced expression of neurodevelopment-related genes and mitigated the adverse effects of bisphenol-A on locomotor behavior and oxidative stress markers in zebrafish larvae	J. Gu et al. [134]
In vitro	Lignin	Assessing the antioxidant properties of LCCs and their ability to scavenge reactive oxygen species	Chemical analysis of ROS scavenging	LCCs exhibited significant ROS scavenging abilities, suggesting their potential applications in mitigating oxidative stress-related conditions	H. Dong et al. [135]
In vitro	Lignin	Neuroprotective effects of Flax Lignan against NMDA-induced neurotoxicity in primary cultured cortical neurons	Primary cultured cortical neurons	Significantly reversed the NMDA-induced increase in the Bax/Bcl-2 ratio, suggesting its potential role in promoting neuronal survival and reducing apoptosis	X.-B. Li et al. [136]
In vitro/In vivo	PLGA	Drug encapsulation for BBB crossing	Combined cell culture and animal studies	Effective distribution of neuroprotective agents with maintained biocompatibility	D.N. Kapoor et al. [137]
In vivo	PLGA	Polysorbate-80-coated PLGA thymoquinone nanoparticles for the treatment of Alzheimer’s disease	Rat model study with cognitive assessment	PLGA nanoparticles were effective carriers for improving thymoquinone delivery to the brain while providing controlled release properties	M. Yusuf et al. [138]
In vivo	Modified BSA	Procyanidin C-1 delivery	Mouse model of AD with behavioral analysis	Improved cognition and reduced amyloid-beta in AD models	L. Duan et al. [139]

AD—Alzheimer’s disease; BAX/BCL-2 ratio—BCL-2-associated X protein/B-cell lymphoma 2 protein ratio; BSA—bovine serum albumin; BBB—blood–brain barrier; CIMP—CPG island methylator phenotype; CXCL12—C-X-C motif chemokine ligand 12; LCCS—lignin–carbohydrate complexes; NGF—nerve growth factor; NMDA—N-methyl-D-aspartate; PC12—rat adrenal pheochromocytoma cell line; PD—Parkinson’s disease; PLGA—poly(lactic-co-glycolic acid); ROS—reactive oxygen species; SH-SY5Y—human neuroblastoma cell line.

## 5. Summary and Conclusions

NDs represent an increasing global health challenge, affecting over 60 million people worldwide and ranking as the seventh leading cause of death [4]. The prevalence of these disorders has shown a dramatic increase, with dementia cases alone rising by 117% between 1990 and 2016 [140]. The complexity of treating these conditions is largely due to the selective nature of the BBB, which prevents approximately 98% of small-molecule medications from effectively reaching the brain [64,66]. This review has examined the potential of biopolymers, particularly chitosan, fish collagen/gelatin, and alginate, in addressing these therapeutic challenges through various neuroprotective mechanisms and drug delivery strategies.

Chitosan has demonstrated significant promise in neuroprotection through multiple mechanisms, including controlled drug release, enhanced antioxidant activity, and metal-ion chelation [91,102]. Studies have shown its effectiveness in delivering dopamine for PD treatment and reducing oxidative stress through the enhancement of antioxidant enzyme activities [92]. The biocompatibility and ability to cross the BBB make chitosan particularly valuable for targeted drug delivery applications, especially in delivering therapeutic agents like curcumin with anti-depressant effects [97,101].

Fish collagen and gelatin have shown noteworthy neuroprotective properties, particularly in reducing the mass of post-traumatic lesions and improving spatial memory in animal models [107]. These biopolymers have demonstrated the ability to stimulate neural plate formation and enhance learning processes in aged mice [110]. Additionally, gelatin has shown promise in treating BBB leaks and reducing inflammation following central nervous system injuries, with smaller peptides of the hydrolysates showing enhanced protective effects on CNS cells [117].

Alginate-based systems have exhibited multiple neuroprotective mechanisms, including anti-inflammatory effects and structural support for tissue regeneration [125]. Particularly promising is the role of PM in improving motor function and preventing dopaminergic neuronal loss in PD research [126]. Recent studies have demonstrated success with polyelectrolytically coated alginate systems for sustained drug delivery and improved outcomes when combined with stem cells and therapeutic agents [127,129].

Emerging research has also highlighted the potential of other biopolymers, such as lignin, PLGA, and glucose-modified BSA/procyanidin complexes. These materials have shown promise in reducing oxidative stress, improving drug delivery across the BBB, and enhancing cognitive function in ND models [134,137,139].

Current therapeutic approaches primarily focus on symptom management rather than disease modification [52]. While treatments like cholinesterase inhibitors and levodopa provide temporary relief, they do not alter disease progression [54]. The emergence of biopolymer-based delivery systems offers new possibilities for more effective therapeutic interventions, particularly in addressing the challenges of drug delivery across the BBB through mechanisms such as carrier-mediated transport, receptor-mediated transcytosis, and adsorptive transcytosis [64,76].

While significant progress has been made in understanding and developing biopolymer-based approaches for treating NDs, further research is needed, particularly in human clinical trials. The majority of current evidence comes from animal studies and in vitro experiments [112,113]. Future investigations should focus on optimizing delivery systems, improving targeting efficiency, and conducting more extensive clinical trials to validate these promising findings in human populations. However, developing effective biopolymer scaffolds for neuroprotection faces several key challenges. The precise control of scaffold degradation, coupled with ensuring the non-toxic degradation of byproducts, is essential for biocompatibility. Translational hurdles remain due to a lack of standardization between preclinical and clinical studies. For CNS applications, the blood–brain barrier and rapid drug degradation after intracerebral injection limit therapeutic efficacy. Current animal models often oversimplify complex neurological disease pathways and poorly reflect the heterogeneity of human conditions. Variability in therapeutic effects across models, combined with limitations in biopolymer mechanical properties and the potential for inflammatory or toxic responses, necessitates further research to optimize scaffolds for neuroprotection [141,142,143].

The collective evidence indicates that biopolymers like chitosan, fish collagen/gelatin, and alginate show therapeutic potential through various neuroprotective mechanisms and enhanced BBB penetration. However, with evidence primarily coming from animal and in vitro studies, extensive clinical trials are needed to validate these findings and address key challenges in scaffold degradation, standardization, and therapeutic delivery.

## Data Availability

No new data were generated.

## Abbreviations

The following abbreviations are used in this manuscript:

AD	Alzheimer’s disease
AKT	protein kinase B
ALS	amyotrophic lateral sclerosis
AMT	adsorptive transcytosis
BAX	Bcl-2-associated X protein
BBB	blood–brain barrier
BCL-2	B-cell lymphoma 2
BSA	bovine serum albumin
CIMP	CPG island methylator phenotype
CMT	carrier-mediated transport
CS NPs	chitosan nanoparticles
CXCL12	C-X-C motif chemokine ligand 12
ECM	extracellular matrix
FTD	Frontotemporal Dementia
GLUT1	glucose transporter 1
HD	Huntington’s disease
IL-1β	interleukin-1 beta
LBD	Lewy body dementia
LCCS	lignin–carbohydrate complexes
MVECs	microvascular endothelial cells
NDs	neurodegenerative disorders
NGF	nerve growth factor
NLRP-3	NOD-like receptor family pyrin domain containing 3
NMDA	N-methyl-D-aspartate
PC12	rat adrenal pheochromocytoma cell line
PD	Parkinson’s disease
PG	polyguluronic acid
PI3K	phosphoinositide 3-kinase
PLGA	poly(lactic-co-glycolic acid)
PM	polymannuronic acid
RMT	receptor-mediated transcytosis
ROS	reactive oxygen species
SH-SY5Y	human neuroblastoma cell line
SLC	solute carrier
